# Prenatal diagnosis of rapidly enlarging choledochal cyst with gastric outlet obstruction

**DOI:** 10.1093/jscr/rjab547

**Published:** 2021-12-31

**Authors:** P Nina Scalise, Alex Yang, Corinne Neumeyer, Antonio R Perez-Atayde, Jamie R Robinson, Heung Bae Kim, Alex G Cuenca

**Affiliations:** Department of General Surgery, Boston Children’s Hospital, Boston, MA, USA; Department of General Surgery, Boston Children’s Hospital, Boston, MA, USA; Department of General Surgery, Boston Children’s Hospital, Boston, MA, USA; Department of Pathology, Boston Children’s Hospital, Boston, MA, USA; Department of General Surgery, Boston Children’s Hospital, Boston, MA, USA; Department of General Surgery, Boston Children’s Hospital, Boston, MA, USA; Department of General Surgery, Boston Children’s Hospital, Boston, MA, USA

## Abstract

Choledochal cysts are congenital malformations of the biliary tract that involve aberrant configurations of the pancreaticobiliary ductal system. The pathology exists on a spectrum from fusiform dilation of the common bile duct to multiple dilations involving the intra- and extrahepatic bile ducts with potential risks of malignant transformation and hepatic fibrosis. Advancements in ultrasound technology have increased the incidence of prenatal diagnosis of choledochal cysts. Here, we present the case of a prenatally diagnosed initially asymptomatic Type I choledochal cyst with rapid progression in the neonatal period to a complete gastric outlet obstruction within the first month of life. We demonstrate the feasibility of cyst resection and reconstruction with Roux-en-Y hepaticojejunostomy in the neonatal age group. Finally, we discuss management of the case based on evolving imaging findings and laboratory evidence of impending liver dysfunction.

## INTRODUCTION

Choledochal cysts (CCs) are rare and usually benign abnormalities consisting of a single or multiple dilations of the intra- or extrahepatic bile ducts. Though the exact cause of these malformations is unknown, a commonly accepted etiology is an anomalous junction between the pancreatic and common bile duct (CBD), resulting in reflux of pancreatic fluid into the CBD leading to weakening of the bile duct wall and cyst formation [1, 2]. While 80% of cases are identified in early infancy and within the first decade of life, advances in fetal imaging have made the prenatal diagnosis of CCs more prevalent [3, 4]. Despite nearly half of prenatally diagnosed patients being asymptomatic, definitive treatment is warranted to mitigate the potential serious complications of biliary infection, cyst rupture or progression to hepatic fibrosis, the latter being associated with fetal and newborn diagnosis [4–6]. We report a case of a prenatally diagnosed Type I CC with rapid growth in the postnatal period causing gastric outlet obstruction (GOO). Close neonatal observation was ultimately followed by delayed cyst excision and Roux-en Y hepaticojejunostomy.

## CASE REPORT

A 41-year-old woman, gravida 7, para 2, aborta 4, had a routine prenatal ultrasound at 28 weeks, 6 days which showed a large fusiform-shaped cyst in the fetal abdomen extending from the porta hepatis to the descending duodenum, concerning for a Type I CC. Fetal MRI on the same day showed the cyst measuring 2.6 × 1.1 × 3.3 cm, along with mildly dilated intrahepatic ducts which appeared to be communicating with the cyst. No other prenatal malformations were observed and aside from a maternal history of idiopathic thrombocytopenic purpura, the pregnancy was otherwise uneventful. A female infant was born at 38 weeks gestational age by cesarean section due to failure to progress and non-reassuring fetal heart tones. Apgar scores upon delivery were 9 and 9 at 1 min and 5 min, respectively. Her physical exam was benign with a soft abdomen and no palpable masses or jaundice. Abdominal ultrasound redemonstrated the cystic malformation, now measuring 3.3 × 2.3 × 6.7 cm with associated intrahepatic biliary ductal dilation. Laboratory results after birth showed total bilirubin (TB) 6.4 and direct bilirubin (DB) 0.3. Prior to discharge from the neonatal intensive care unit on day of life (DOL) one, the parents were informed of a need for subsequent surgical intervention, though not in the emergent setting given the infant’s lack of symptoms.

**Figure 1:**
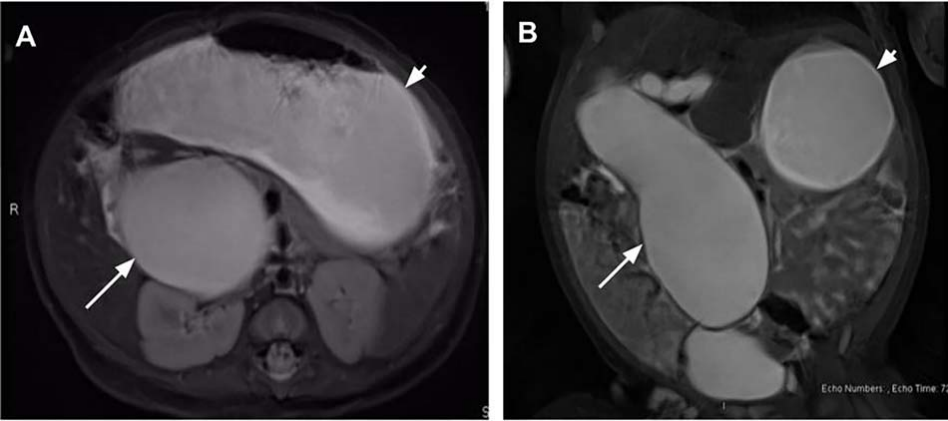
Magnetic resonance cholangiopancreatography on day of life (DOL) 36 in coronal **(A)** and axial **(B)** views showing a massive fusiform dilatation of the common bile duct (long arrow) and markedly distended stomach (short arrow) consistent with gastric outlet obstruction.

On DOL 22, the patient presented for a routine outpatient follow-up. She was tolerating feeds and stooling normally at home; her exam was unremarkable aside from mild abdominal distention. Laboratory results at that time showed TB 1.6 (0.3–1.2) and DB 0.8 (0–0.4). An magnetic resonance cholangiopancreatography (MRCP) was subsequently obtained on DOL 36 which revealed progression of the cyst, now measuring 5.1 × 4.2 × 10.8 cm, associated with a markedly distended stomach and decompressed small bowel (Fig. 1). The cyst was also observed to be displacing loops of bowel medially and inferiorly, exerting mass effect on the right kidney and extending into the pelvis where it was abutting the bladder. The patient was subsequently referred to the emergency department for admission and operative repair. Due to significant increase in size of the cyst on MRCP with impending GOO and cholestatic lab pattern (ALP 466, TB 2.1, DB 1.6), the patient was admitted for open CC excision at DOL 39. Exam on admission was significant for mild scleral icterus and abdominal distension with reported acholic stools. Notably, she was still tolerating feeds with no episodes of emesis.

**Figure 2:**
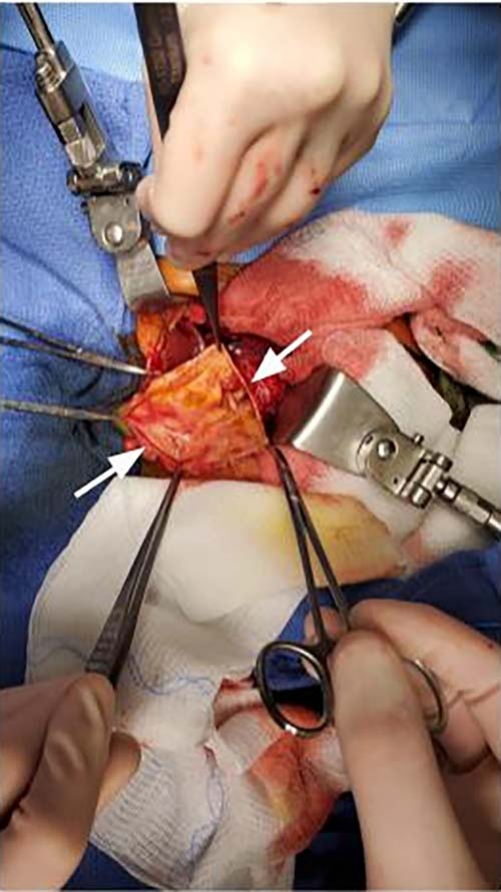
Intraoperative image showing incised cyst wall (arrows).

**Figure 3:**
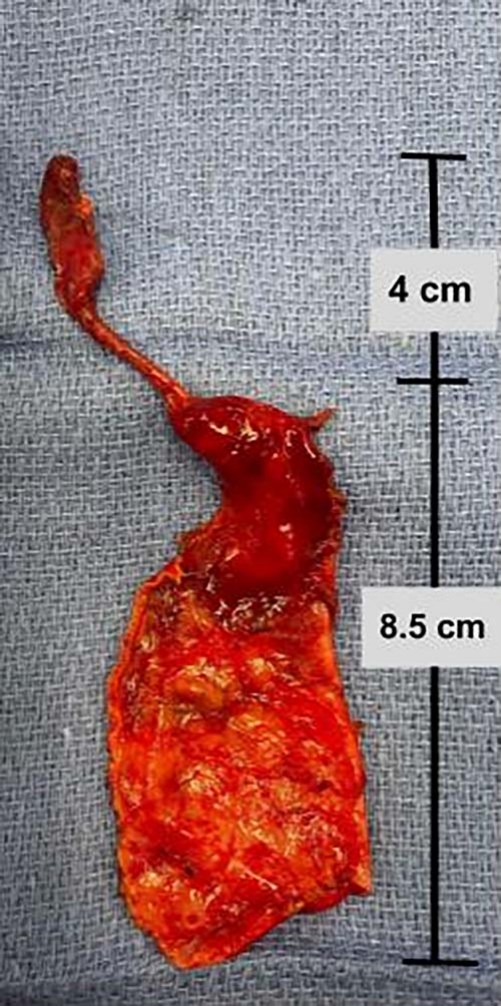
Complete excised gross specimen comprising choledochal cyst wall and gallbladder.

**Figure 4:**
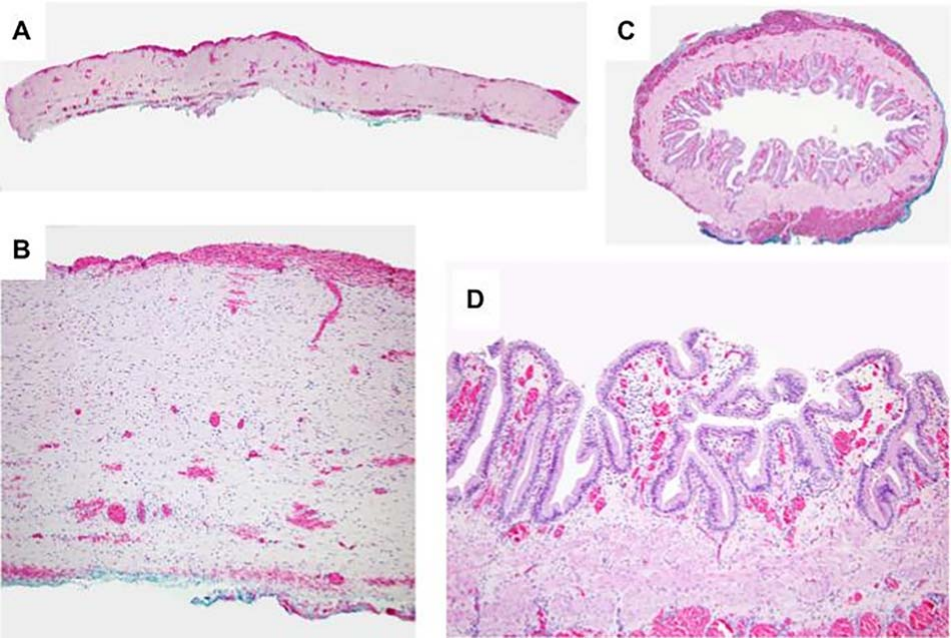
Light microscopy of resected specimen. **A.** Cross section of the choledochal cyst wall showing entirely denuded surface with absent mucosa. The surface of the cyst wall was congested and hemorrhagic without inflammation. **B.** The cyst wall is composed of quiescent, uniform fibrocytes. **C.** A cross section of the mildly dilated CBD with preserved intact mucosa. **D.** The biliary epithelium at higher power is well-developed and lacks atypia.

Intraoperatively, a large cystic structure, noted to be the CC, was identified spanning the right upper and lower quadrants down to the pelvis, displacing surrounding tissue. Following the cholecystectomy, the CC was freed circumferentially, identifying and preserving the right hepatic artery. Once the cyst was able to be elevated off of the portal structures, the decision was made to drain the cyst. The cyst was entered at the level of the common hepatic duct below the bifurcation, and significant bilious sludge was aspirated. Once the cyst was drained, it was excised from just proximal to the bifurcation of the left and right hepatic ducts to the distal CBD which was oversewn after clear identification of the pancreatic duct (Fig. 2). Reconstruction was performed with a Roux-en-Y hepaticojejunostomy with a retrocolic 45 cm Roux limb. A 10-French drain was left in the right upper quadrant and the abdomen was closed. The final excised specimen is shown in Fig. 3. Microscopically the cyst surface was entirely denuded with absent mucosa, the wall was composed of quiescent, uniform fibrocytes. The superficial zone of the cyst wall was congested and hemorrhagic Fig. 4A-4B. The CBD was mildly dilated with intact mucosa (Fig. 4C-4D). There were no apparent complications and the patient tolerated the procedure well. The postoperative course was notable for asymptomatic bradycardia that self-resolved. Feeds were initiated on the fourth postoperative day, surgical drain was removed on the sixth postoperative day, and the patient was ultimately discharged home on the seventh postoperative day. Follow-up ultrasound within 1 week of discharge showed typical postoperative changes without evidence of fluid collection or biliary ductal dilation, and normal appearance of the liver without evidence of fibrosis. LFTs also normalized (TB 0.7 mg/dl and DB 0.2 mg/dl). The patient was most recently evaluated in clinic at 8 weeks of age at which time she was feeding and stooling appropriately.

## DISCUSSION

CCs are congenital malformations of the biliary tract that involve aberrant configurations of the pancreaticobiliary ductal system. The most widely accepted classification was proposed by Todani and colleagues in 1977 and describes five types of CC: Type I as a fusiform or cystic dilation of the CBD, Type II as a true diverticulum of the CBD, Type III as a choledochocele protruding into the duodenum, Type IV as dilation of the intra- and extrahepatic bile ducts and finally Type V, or Caroli’s disease, as fusiform or saccular dilation of intrahepatic ducts [3]. While improvements in ultrasound technology have made the prenatal diagnosis of CCs more common, a move toward surgical management of asymptomatic patients in the immediate postnatal period has been forestalled by concerns over surgical and anesthetic complications in the neonatal stage [4]. A common and acceptable management strategy for asymptomatic patients is careful monitoring, followed by elective surgery at 3–6 months of age [2]. Our case is unusual due to the particularly rapid cyst development leading to complete GOO in the early neonatal period. At birth, the patient was completely asymptomatic with normal liver function laboratory studies, which led to the decision to conservatively monitor her with a plan for eventual definitive excision. However, at her first routine follow-up at DOL 22, she was found to have elevated bilirubin and GGT which prompted an MRCP that showed significant cyst progression and GOO.

Hamada et al. [7] previously reported a case in which a patient with a large prenatally diagnosed CC was found to have GOO at birth. This patient quickly became intolerant of feeds due to rapid cyst growth in the first few days of life. Her symptoms warranted percutaneous drainage of the cyst at DOL 4, followed by delayed excision at DOL 81 [7]. Yurttutan et al. [8] reported a prenatally diagnosed type 1a CC measuring 16 cm at the time of operation, though without evidence of GOO; the cyst was excised with Roux-en-Y hepaticojejunostomy. In our case, we elected for surgical excision alone rather than temporizing drainage, given the age at which she presented. Interestingly, unlike the patient reported by Hamada et al., our patient presented without overt feeding intolerance or emesis, which may suggest that dilation of the cyst over the course of a month may have caused enough chronic gastric dilation to mask acute GOO symptoms.

A previous review by Lugo-Vicente et al. [5] showed that progressive cyst growth in the neonatal period was present in 56% of patients with prenatally diagnosed CCs. While the etiology of this growth has not been thoroughly studied, many have proposed that increased bile and pancreatic juice production while the infant is milk feeding may accelerate certain pathophysiological processes in cyst development [7, 9]. Nevertheless, despite the prevalence of cyst growth among patients with CCs, growth itself is not commonly a surgical indication and there have been no studies to-date to show the implications of growth rate on future complications [9].

This case highlights the challenge in management of asymptomatic patients with progressive CC growth. Indolent growth without any overt symptoms may mask the potential severity of the disease and patients may present *in extremis* [10]*.* Liver dysfunction is not always apparent and requires laboratory confirmation [11]. Because most prenatally diagnosed CCs have a propensity to grow during the neonatal period and ultimately result in a degree of liver dysfunction, frequent imaging and laboratory testing should be performed in asymptomatic patients with CCs to effectively assess disease progression and plan for potential intervention.

## CONFLICT OF INTEREST STATEMENT

None declared.

## FUNDING

None.
